# The Bidirectional Relationship between Body Weight and Depression across Gender: A Simultaneous Equation Approach

**DOI:** 10.3390/ijerph18147673

**Published:** 2021-07-19

**Authors:** Jun Zhang

**Affiliations:** School of Agricultural Economics and Rural Development, Renmin University of China, No. 59 Zhongguancun Ave., Beijing 100872, China; junzhang@ruc.edu.cn

**Keywords:** body weight, obesity, depression, simultaneous equation system, gender difference

## Abstract

Purpose: This study investigates the bidirectional relationship between body weight and depression for both males and females in the U.S. Methods: Data are drawn from the 2019 Behavioral Risk Factor Surveillance System (BRFSS), and a simultaneous ordered probability system is estimated with maximum likelihood estimation (MLE) to accommodate the two-way causality between depression and body weight categories. The variable of depression is measured by individuals’ past depressive records and current mental health status. Results: Depression and body weight are found to affect each other positively for both males and females on average. In a randomized population, the results of average treatment effects suggest significant body weight differences between depressed and non-depressed individuals. Age and other sociodemographic factors affect body weight differently between genders and between the people with depression and those without. Conclusion: The positive bidirectional relationship between body weight and depression is found. The effect of depression on body weight is significant among both males and females in a randomized population, and females who experience depression are most likely to be obese and less likely to have normal weight compared to females without depression. The risks of overweight and obesity are high among people who are less educated or unable, who have poor health statuses, and who had high blood pressure.

## 1. Introduction

Obesity and depression are two major public health concerns involving a huge population around worldwide. More than 350 million people of all ages suffered from depression during 2012 in all regions of the world [1], and it is estimated that 1 out of 20 people reported having an episode of depression in the previous year worldwide [2]. In 2014, more than 1.9 billion adults were overweight around the world and over 600 million of them were obese [3]. In the U.S., over one-third of adults and 17% of children were obese during 2011–2012 [4]. Obesity is found to be associated with not only physical ailment but also mental disorders, such as mood disorder, major depression, and anxiety [5]. Existing studies have suggested a linkage between depression and obesity [6,7,8], and a meta-analysis of 19 studies suggested a bidirectional relationship between depression and obesity [9]. However, it is not clear how depression and body weight affect each other simultaneously, and few studies have applied large representative nationwide data to explore such effects.

It is not well understood how obesity impacts mental disorders in the general population; some literature have suggested that depressive symptoms can be caused by negative body image, which is the result of obesity [10,11]. In this sense, people who are obese are more likely to be depressed. It is well documented that obesity presents a risk factor for a wide range of chronic conditions, including cardiovascular disease, cancer, type 2 diabetes, musculoskeletal disease, and pain [12,13], which results in comorbidity with chronic diseases and depression [14]. Therefore, from the biological perspective, one mechanism explaining the relationship between obesity and depression is the pro-inflammatory cytokines that are caused by adipocytes and inflammatory diseases, which can directly influence brain physiology and can contribute to the development of depression [15,16,17,18]. For depression, previous studies suggest that depression may impact body weight, for example through changing eating patterns or physical activity level [19]. Even though depression and obesity issues have been fully investigated separately around the world, most existing studies either concentrate on clinic research with limited samples or exclude important sociodemographic factors. More importantly, few have explored the mutual causality between depression and body weight, with rare exceptions [20,21].

For the impact of body weight on depression, findings from existing studies are not consistent. Some studies found relatively weak evidence supporting the hypothesis that body weight affects depression [7], suggesting that the impact of obesity on depression is insignificant. Other studies found that people who are obese are more likely to be depressed among the general population [5,22,23,24]. For example, one study found the prevalence of depression to be highest among persons with severe obesity [22], and another study found that obesity increases the risks of depressive symptoms [24]. Although previous studies have concentrated on the relationship between obesity and depression among different individual groups [25,26,27], in-depth quantitative analyses of how body weight affects depression across genders are still limited. In addition, most of these studies used logit models and reported odds ratios that can dramatically overstate the relative risk for common outcomes [28].

For the impact of depression on body weight, evidence has been found that depression may impact obesity [23,29,30,31,32]. Although depression can result in weight gain through physical or behavioral mechanisms [33,34], findings of this association are not consistent [29,35,36]. Moreover, previous research on the impact of depression on body weight are mostly restricted to the younger population [20,32] and older individuals have not been the focus of investigations.

The relationship between depression and body weight can be confounded by sociodemographic factors [5,8]. Mechanisms to cope with depressive symptoms and obesity are likely to vary across social and cultural environments [20]. In particular, previous research suggested that gender difference has significant impacts on depression and body weight, with strong evidence that females are more likely to be depressed than males [37,38]. Furthermore, it has been found that obesity is more likely to be associated with depression among females than males [39,40,41]. For example, one study suggested that the prevalence of depression is more than twice as great among females with BMI of 30 or more compared to those with BMI less than 30 [23].

The main research goal of this study is to investigate the bidirectional relationship between depression and body weight. By focusing on the large representative data in the U.S., this study has the following specific objectives: (i) to explore gender differences in the relationship between depression and body weight, (ii) to explore how these bidirectional relationships vary by sociodemographic factors, and (iii) to quantify the effects of sociodemographic characteristics on depression and obesity.

## 2. Materials and Methods

### 2.1. Data and Participants

The research data were drawn from the 2019 Behavioral Risk Factor Surveillance System (BRFSS) collected by state health departments in collaboration with the U.S. Centers for Disease Control (CDC). The BRFSS conducts health-related telephone surveys that collect state data about U.S. residents regarding their health-related risk behaviors, chronic health conditions, and use of preventive series. It covers all 50 U.S. states as well as the District of Columbia and three U.S. territories, which has more than 400,000 adult interviews each year, making it the largest health survey system in the world. The survey participants were randomly selected adults in households, and all responses were self-reported.

### 2.2. Measures

In the 2019 BRFSS data, no indicators for current depressive symptoms were provided, so two questionnaire items were combined to form a proxy for current depressive symptoms. These two items were “(Ever told) (you had) a depressive disorder (including depression, major depression, dysthymia, or minor depression)?”, and “How many days during the past 30 days was your mental health not good?” From responses to these two questions, a binary indicator of current depression status was created. The value of depression equals one if the individual responded “Yes” to the first question and “>0” to the second question, and zero if the individual responded “No” to the first question and “0” to the second question. In addition, to make sure individuals were correctly assigned to the “depression” and “non-depression” groups, respectively, we excluded individuals who answered “Yes” to the first question and “No” to the second question, and vice versa from our study samples. Individuals who were excluded from the study accounted for less than 20% of the total samples.

The body weight status was measured by the body mass index (BMI), which was calculated as the ratio of height and weight. The raw BMI score recorded in the BRFSS data was coded into four categories: underweight (BMI < 18.5), normal weight (18.5 ≤ BMI < 25), overweight (25 ≤ BMI < 30), and obese (BMI ≥ 30). Removing observations with missing values for important variables led to a sample size of 142,637, among which 71,603 were males and 71,034 females. The frequency distribution of depression and body weight across gender is presented in Table 1.

### 2.3. Sociodemographic Factors

The sociodemographic variables included age, income, race, education, and dummy variables indicating home ownership, employment status, and marital status. Table A1 of Appendix A presents the definitions and sample statistics of explanatory variables used in this paper. Annual household income was coded into eight categories, where 1 denotes the lowest and 8 denotes the highest categories. In addition, individual’s health statuses are posited to affect depression. Identification of the simultaneous equation model requires the use of instruments in the two outcome equations; therefore, three dummy variables indicating “very good or excellent”, “good”, and “fair or poor” of self-reported health status were used in the equation for depression but not body weight, whereas fruit-eating frequency and previous high blood pressure records were used solely in the body weight equation. The data for fruit-eating frequency were coded from responses to the BRFSS question: “Total fruits consumed per day?” Previous studies found that an increased consumption of fruits and vegetables is inversely related to body weight and that the benefits are greater for fruits compared to vegetables [42,43]. For this reason, we included fruit-eating frequency as the dietary control variable in the body weight equation. Obese people usually have high blood pressure; thus, if the individual has high blood pressure records, it is very likely that the individual was obese in the past. Here, we assumed past body weight correlates with current body weight, and high blood pressure records were taken as a proxy for past body weight. In particular, a record of high blood pressure is a dummy variable that equals 1 if the individual has ever been told they have high blood pressure by a doctor, nurse, or health professional. Physical activity has been found to affect body weight and to ameliorate depressive symptoms [38]; thus, a variable that measures physical activity frequencies was also used. Such a physical activity variable reflects the number of times an individual performed physical activity during the last 30 days.

### 2.4. Methods

A two-equation simultaneous system was used to explore the bidirectional relationship between ordinal body weight category ($y_{bmi}$) and binary depression indicator ($y_{depression}$). Details about the model specification of simultaneous system equation are described on pages 346–365 of Williams Greene’s econometrics textbook [44]. In this study, the model is characterized by structural equations for corresponding latent variables ($y_{bmi}^{*}$) and ($y_{depression}^{*}$):

(1) $$y_{bmi}^{*}=\gamma_{1}y_{depression}^{*}+x^{\prime }\alpha_{1}+z^{\prime }\alpha_{2}+u_{1}$$

(2) $$y_{depression}^{*}=\gamma_{2}y_{bmi}^{*}+x^{\prime }\beta_{1}+w^{\prime }\beta_{2}+u_{2}$$

where Equation (1) is used to measure the impact of depression on body weight and Equation (2) is used to measure the opposite. The bidirectional association or mutual causality relationship between body weight and depression is assessed by estimating the above equations simultaneously as a system. Previous studies have applied simultaneous system equations to investigate the bidirectional relationship between subjective well-being and leisure [45]. For the two-equation simultaneous system defined above, $\left(x,z,w\right)$ are vectors of exogenous variables with conformable parameter vectors $\left(\alpha_{1,}\beta_{1},\alpha_{2},\beta_{2}\right),$ and $\left(\gamma_{1},\gamma_{2}\right)$ are scalar parameters. The error terms $\left(u_{1},u_{2}\right)$ are assumed to be distributed as standard bivariate normal with correlation ρ. With ordinal body weight and binary depression categories, the latent variables $\left(y_{bmi}^{*},y_{depression}^{*}\right)$ are mapped to the observed variables $\left(y_{bmi},y_{depression}\right)$ such that

(3) $$y_{bmi}=k\;\mathrm{if}\;\xi_{k-1}<y_{bmi}^{*}<\xi_{k},\;k=0,1,\ldots ,K$$

(4) $$y_{depression}=1\;\mathrm{if}\;y_{depression}^{*}>0\;=0\;\mathrm{if}\;y_{depression}^{*}\leq 0$$

where ξ is the threshold parameter such that $\xi_{0}=-\infty ,\xi_{1}=0,\xi_{K}=\infty$ and $\left(\xi_{2},\ldots ,\xi_{K-1}\right)$ are estimable.

The above simultaneous equation system can be estimated using the two-step estimation method proposed by Maddala [46], whose estimators are statistically consistent but inefficient. In this study, a more efficient full information maximum-likelihood (FIML) method is applied. To facilitate an interpretation of the effects of depression on body weight categories, average treatment effects of binary depression on ordinal body weight are calculated. In addition, since the simultaneous model proposed above is a nonlinear system model, marginal effects of explanatory variables must be used to interpret the effects on outcome variables. For statistical inference, standard errors of the treatment effect and average marginal effects are calculated using a mathematical approximation procedure known as the delta method. Derivations of the log-likelihood function, average marginal effects, and treatment effects of depression on body weight are presented in the Appendix A.

## 3. Results

We first tested if there is a relationship between body weight and depressing using a chi-square test. The test results suggest that there is a statistically significant relationship between body weight and depression for males ($\chi^{2}=264.43$, df = 3, *p*-value < 0.001) and females ($\chi^{2}=1234.84$, df = 3, *p*-value < 0.001). We then determined whether to estimate the model with separate male and female samples or a pooled full sample. The statistical test was carried out with a likelihood ratio (LR) test, which is similar to the Chow test in linear regression models. Specifically, we defined the maximum log-likelihood values for the male, female, and pooled full samples as $\log L_{m}$, $\log L_{f}$, and $\log L_{p}$ with corresponding numbers of parameters $k_{m}$, $k_{f}$, and $k_{p}$, such that a gender dummy variable in both equations was used for the pooled sample. Then, under the null hypothesis that the slope parameters are equal between genders, the likelihood-ratio (LR) statistics $LR=2\left(\log L_{m}+\log L_{f}-\log L_{p}\right)$ was chi-square distributed with $k_{m}+k_{f}-k_{p}$ degrees of freedom (df). Using results from the three samples, the hypothesis of equal slope coefficients is rejected (LR = 3590.134, df = 39, *p*-value < 0.001), suggesting separate estimation with the male and female samples.

The FIML estimates by gender are presented in Table A2 of Appendix A, and the results are summarized here, which reveal the average impacts of each variable on two outcome variables. On average, the endogenous depression has a positive and significant coefficient in the body weight equation for both males (0.472) and females (0.485), while body weight has a positive and significant coefficient in the depression equation for males (0.407) and females (0.317). The positive two-way association between depression and body weight suggests that males and females with depression are more likely to be heavier than their non-depressed counterparts and that males and females who are heavier are at a higher risk of depression on average.

Of the 18 exogenous variables in the depression equation, 15 are significant at the 10% level for males and 17 variables are significant for females. The two health status variables are significant in the depression equation at the 1% level of significance in both samples, rejecting the hypothesis of weak instruments and justifying the use of the variables for identification. Of the 18 exogenous variables in the body weight equation, 16 are significant at the 10% level for males and 15 are significant for females. The coefficient of high blood pressure record is positive and significant among both males and females, again rejecting weak instruments. The estimates also differ greatly among males and females, in terms of signs, magnitudes, and statistical significance.

As discussed earlier, to further exploit the effects of depression and explanatory variables on different category of body weight, the average treatment effects and marginal effects of explanatory variables must be calculated in order to interpret meaningful results from the nonlinear two-way simultaneous equation system model. The average treatment effects (ATE) of the binary depression on ordinal body weight were calculated from the FIML estimates. The results are presented in Table 2. Table 3 and Table 4 presents marginal effects on the joint probabilities of depression and body weight categories for male and female samples, respectively. Interpretations of the treatment and marginal effect results are discussed below.

## 4. Discussion

### 4.1. Average Treatment Effects of Depression on Body Weight

For both males and females, ATEs are significant for all four body weight categories, suggesting significant body weight differences between people with and without depres-sion in a randomized population. For a randomly selected male, compared to someone without depression, a male with depression has 0.07, 1.00, and 0.07 percentage point higher probabilities to be underweight, normal weight, and overweight, respectively, while the probability is lower by 1.14 percentage point for obesity. For a randomly selected female, compared to the someone without depression, a female with depression has a 0.63 percentage point lower probability to be underweight and 5.01 percentage point lower probability of having normal weight. The probabilities are higher by 0.54 percentage points for overweight and by 5.11 percentage points for obesity. This higher probability of obesity among females with depression is consistent with the findings by others [32]. The results from ATE suggest that depression plays different roles in affecting body weight categories across gender and that, for individuals who are depressed, males are less likely to be obese and females are more likely. These findings are more informative than previous studies, which implicitly assumed that the impact of depression is the same for all body weight categories [20,21].

### 4.2. Marginal Effects of Explanatory Variables for Males

Age affects body weight between both the people with and without depression. Among males without depression, a 10-year increase in age is associated with 0.04, 1.35, 1.61, and 0.30 percent point increases in the probabilities of being underweight, normal weight, overweight, and obese, while among people with depression, a 10-year increase in age is associated with 0.02, 0.75, 1.54, and 0.99 percentage point decreases in the probabilities of being underweight, normal weight, overweight, and obese, respectively. This finding suggests that, for males, with an increase in age, (i) males without depression are more likely to be overweight and less likely to be underweight and (ii) males with depression are more likely to be underweight and less likely to be overweight.

Income affects body weight differently among people with and without depression. For example, an increase in income increases the probability of obesity by 1.16 percentage point for males without depression, but it decreases the probability of being obese by 0.16 percentage point for males with depression. The signs of exercise are as expected for underweight and obese males, but the magnitudes are small. Race affects some body weight categories among both males with and without depression. For example, compared to males of other races with depression, a black person has 0.03, 0.84, and 0.98 percentage point lower probabilities to be underweight, normal weight, and overweight, respectively. Education affects males with and without depression differently. Compared with males without depression with only a high school diploma, those with a bachelor’s degree have a 0.20 (3.38) percentage point higher probability to be underweight (normal weight) and a 1.53 (6.36) percentage point lower probability to be overweight (obese), but their depressed counterparts have 0.05, 1.47, 2.04, and 0.76 percentage point higher probabilities to be underweight, normal weight, overweight, and obese, respectively. The effects of employment are stronger among males with and without depression. Specifically, employed males without depression are most likely to be obese and least likely to be normal weight compared to their unemployed counterparts.

Home ownership and the ability to work have opposite effects on males with and without depression in terms of being overweight and obese. For example, compared to a male who is able to work, a male without (with) depression who is unable to work has 6.65 and 7.13 (2.36 and 6.21) percentage point lower (higher) probabilities to have normal weight and to be overweight, suggesting that males with depression who are unable to work are more likely to have normal weight and to be overweight than their non-depressed counterparts.

Regarding marital status, compared with their single counterparts, married males without depression are least likely to be normal weight and most likely to be obese. In particular, married males without (with) depression have 1.32 and 8.18 (1.78 and 0.36) percentage point higher (lower) probabilities to be overweight and obese, suggesting the reciprocal relation of marriage among overweight (obese) males with and without depression.

Self-reported health status affects body weight, with very good health and poor health conditions playing opposite roles in affecting body weight. Compared to males with depression in good health status, males in very good or excellent (poor) health status have 0.01, 1.23, 3.71, and 3.18 (0.00, 0.87, 2.81, and 2.55) percentage point lower (higher) probabilities of being underweight, normal weight, overweight, and obese. These results suggest that males with depression and with very good or excellent health status are less likely to be overweight and obese, while males with depression and with relatively poor health status are more likely to be overweight and obese. The number of total fruits consumed per day plays some roles in affecting body weight. For example, a one-unit increase in daily fruit consumption decreases the probability to be obese by 0.48 (0.07) percentage point for males without (with) depression. A high blood pressure record has expected signs in affecting body weight and much greater magnitudes among males without pression. Specifically, a record of high blood pressure is associated with a 0.61 (13.00) percentage point lower probability to be underweight (normal weight) and 14.13 percentage point higher probability to be obese among males without depression.

### 4.3. Marginal Effects of Explanatory Variables for Females

Similar to results for males, age affects body weight differently between females with and without depression. For a female without depression, a 10-year increase in age increases (decreases) the probability of being overweight by 2.49 (2.70) percentage points if she is non-depressed (depressed), suggesting a positive (negative) role of age in body weight.

Considering the effect of income on body weight, we found that an increase in income increases the probabilities of being underweight, normal weight, and overweight by 0.04, 0.59, and 0.25 percentage point among people without depression. As expected, physical activity or exercise reduces body weight somewhat among both people with and without depression.

Unlike their male counterparts, race has larger effects on body weight for both black and Hispanic females, especially among those without depression. For instance, compared with females of other races without depression, being black decreases the probability of being normal weight by 10.57 percentage points but increases the probability of obesity significantly by 16.26 percentage points, which suggests that black females without depression have a much higher (lower) probability of being obese (normal weight). Compared with females of other races with depression, being white increases the probabilities of being underweight, normal weight, overweight and obese by 0.06, 2.51, 3.61, and 3.22 percentage points, suggesting that white females with depression are more likely to be overweight and obese.

The effects of education on females are similar to those on males. Compared with females without depression with only high school education, females with a college degree are more likely to have normal weight and less likely to be overweight and obese. Among those with depression, females with a college degree have 0.15, 3.34, 2.34, and 0.91 percentage point higher probabilities of being underweight, normal weight, overweight and obese, respectively, than those with only a high school diploma. This finding suggests that, among females with depression, college-educated individuals are most likely to have normal weight and overweight, and least likely to be underweight and obese. Regarding employment status, employed females who are non-depressed have a 2.70 percentage point higher probability to be normal weight, while females with depression have a 4.16 percentage point lower probability to be obese.

The ability to work has similar effects on the body weight for females to those for males. For example, a female without (with) depression and is unable to work has 8.08 (8.34) and 5.42 (8.04) percentage point lower (higher) probabilities of being overweight and obese than their peers who are able to work, suggesting that females with depression who are unable to work are at a higher risk of being overweight and obese compared with their non-depressed counterparts. Homeownership status is positively associated with body weight for females without depression, but negatively associated with that of females with depression. In terms of marital status, we find that, compared with single or separated females, married females without depression are least likely to be underweight and most likely to be obese, while married females with depression are least likely to be normal weight and most likely to be underweight.

Self-reported health status plays similar roles in affecting body weight of females to those of males, but the effects are greater in females with depression in terms of magnitudes. These results suggest that females with very good or excellent health status are least likely and those with poor health are most likely to be obese. In particular, we find that healthy females more likely to be normal weight than females with depression who report excellent or very good health status. A high blood pressure record has expected signs and similar effects on females to those on males, and it has much stronger impacts on the body weight of females without depression than those of their depressed counterparts.

### 4.4. Implications for Clinical Practice and Public Health

As a key finding, our study emphasized the positive bidirectional relationship between depression and body weight in the general population, and such relationship across gender. Due to the positive association between depression and body weight, clinical practitioners need to pay particular attention to obese individual’s mental health status and depressed individual’s body weight status. For the treatment of depression among the general population, supplementation, which is both good for alleviating depressive symptoms and reducing body weight, should be considered. For example, the magnesium supplementation has been found to be beneficial in both depression alleviation and body weight losing [47,48]. In addition, existing studies also suggest that dietary fiber can reduce depressive symptom [49] and body weight [50]. However, our results suggest differential effects of the interaction between depression and body weight across gender and sociodemographic factors. Though depression is positively associated with an increase in body weight among both males and females, compared with their non-depressed counterparts, males with depression are found least likely to be obese and females with depression are found most likely to be obese. In this sense, clinical practitioners should treat males and females with depression differently. For males with depression, the comorbidity of obesity is less a concern, while for their female counterparts, the risk of obesity must be seriously considered. The finding of bidirectional relationship between body weight and depression suggests that policy measures should be deliberated with such a causality in mind. The differential effects of the interaction between depression and body weight and of the roles of sociodemographic characteristics in these public health outcomes between genders and between the depressed and non-depressed suggest that there is no uniform approach to the amelioration of depression and obesity issues. The most effective public health intervention to combat depression, and overweight and obesity might consist of a portfolio of systemic and targeted interventions designed to address the health burdens of specific genders within various mental health groups.

### 4.5. Limitations

This study has several limitations. First, it was conducted based on the cross-sectional data. Although cross-sectional evidence is informative, it does not provide detailed insight into the exact mechanisms linking depression and obesity [51]. It could be possible that individuals with depression gain body weight gradually over time, whereas it is also possible that obesity leads to depression over time through negative self-image. In this case, longitudinal and panel data should be used to investigate the long-term effects between obesity and depression, which can reduce estimation bias. Second, it was restricted to reginal populations in the U.S. With data from only one country, the effects of culture and lifestyle on depression and obesity cannot be explicitly uncovered. Lastly, the measure for depression was constructed from self-reported questions rather than individuals’ actual diagnosis. Measures based on self-reported questions may yield reporting errors and eventually affect research results.

## 5. Conclusions

There exist numerous studies on the determinants of mental health and body weight, but there is a dearth of information on interactions between the two important public health issues. Gender differences in association between mental health and body weight and in the sociodemographic determinants of the two have also remained under-explored. This study attempts to fill this gap of knowledge in the empirical literature. We used the most recent national data from the U.S. Empirical analysis was carried out by estimating a simultaneous ordered probability model with ordinal body weight categories and binary depression status.

Our estimates suggest a bidirectional relationship between depression and body weight among both males and females. Average treatment effects of depression on body weight suggest significant body weight differences between individuals with and without depression, and females with depression are most likely to be obese and least likely to have normal weight compared with their non-depressed counterparts.

We find that sociodemographic characteristics play differential roles in body weight and depression between males and females and between those with and without depression. Age is negatively associated with body weight among those with depression and positively associated with those without depression for both males and females. The risks for being overweight and obesity are high among the less educated and in those unable to work, with poor health, and with a record of high blood pressure.

This study is among the first to evaluate the two-way bidirectional relationship between depression and body weight of the general population across gender and major sociodemographic factors with large representative national data. The finding of a bidirectional relationship between depression and body weight suggests that policy measures should be designed with such a causality in consideration. Further studies might consider the use of longitudinal data and might investigate these issues among various sub-population, such as teenagers, minorities, and the disabled.

## Figures and Tables

**Table 1:** Frequency distribution of depression and body weight categories.

	Body Weight Category				
Depression Status	Under	Normal	Over	Obese	Total
	Male				
Non-depressed	385 (0.6%)	15,434 (24.3%)	28,790 (45.4%)	18,820 (29.7%)	63,429 (100%)
	(76.2%)	(87.6%)	(90.5%)	(86.9%)	(88.6%)
Depressed	120 (1.5%)	2187 (26.8%)	3034 (37.1%)	2833 (34.7%)	8174 (100%)
	(23.8%)	(12.4%)	(9.5%)	(13.1%)	(11.4%)
Total	505 (0.7%)	17,621 (24.6%)	31,824 (44.5%)	21,653 (30.2%)	71,603 (100%)
	(100%)	(100%)	(100%)	(100%)	(100%)
	Female				
Non-depressed	911 (1.7%)	21,375 (39.2%)	18,253 (33.4%)	14,049 (25.7%)	54,588 (100%)
	(73.9%)	(81.7%)	(79.1%)	(68.4%)	(76.9%)
Depressed	322 (2.0%)	4795 (29.1%)	4830 (29.4%)	6499 (39.5%)	16,446 (100%)
	(26.1%)	(18.3%)	(20.9%)	(31.6%)	(23.1%)
Total	1233 (1.8%)	26,170 (36.8%)	23,083 (32.5%)	20,548 (28.9%)	71,034 (100%)
	(100%)	(100%)	(100%)	(100%)	(100%)

**Table 2:** Average treatment effects of depression on the probabilities of body weight categories for males and females.

Body Weight Category.	Males	Females
Underweight	0.071	−0.633
	(0.031) **	(0.045) ***
Normal weight	1.000	−5.010
	(0.433) **	(0.360) ***
Overweight	0.069	0.537
	(0.024) ***	(0.033) ***
Obese	−1.140	5.106
	(0.487) **	(0.375) ***

All effects on probabilities are multiplied by 100. Asymptotic standard errors are in parentheses. *** *p* < 0.01, ** *p* < 0.05.

**Table 3:** Marginal effects of explanatory variables on the joint probability of depression and body weight categories for male sample.

	Non-Depressed and				Depressed and			
Variable	Underweight	Normal	Overweight	Obese	Underweight	Normal	Overweight	Obese
Continuous explanatory variables								
Age/10	0.04	1.35	1.61	0.30	−0.02	−0.75	−1.54	−0.99
	(0.01) ***	(0.10) ***	(0.04) ***	(0.11) ***	(0.00) ***	(0.02) ***	(0.04) ***	(0.03) ***
Income	−0.03	−0.58	0.31	1.16	−0.01	−0.28	−0.40	−0.16
	(0.00) ***	(0.08) ***	(0.03) ***	(0.09) ***	(0.00) ***	(0.02) ***	(0.03) ***	(0.02) ***
Number of fruits	0.02	0.46	0.09	−0.48	0.00	0.02	−0.04	−0.07
	(0.00) ***	(0.05) ***	(0.01) ***	(0.05) ***	(0.00) ***	(0.00) ***	(0.01) ***	(0.01) ***
Binary explanatory variables								
Exercise	0.00	0.09	0.02	−0.09	0.00	0.00	−0.01	−0.01
	(0.00) ***	(0.01) ***	(0.00) ***	(0.01) ***	(0.00) ***	(0.00) *	(0.00) **	(0.00) ***
White	−0.16	−3.56	−1.81	2.08	0.01	0.66	1.62	1.18
	(0.03) ***	(0.46) ***	(0.15) ***	(0.50) ***	(0.00) ***	(0.09) ***	(0.15) ***	(0.09) ***
Black	−0.20	−4.67	−0.36	7.18	−0.03	−0.84	−0.98	−0.10
	(0.02) ***	(0.56) ***	(0.31)	(0.82) ***	(0.00) ***	(0.10) ***	(0.22) ***	(0.18)
Hispanic	−0.19	−4.40	−0.37	6.53	−0.02	−0.74	−0.79	−0.00
	(0.02) ***	(0.54) ***	(0.28)	(0.76) ***	(0.00) ***	(0.10) ***	(0.21) ***	(0.16)
<High school	0.17	3.03	−0.14	−3.64	0.02	0.45	0.27	−0.16
	(0.04) ***	(0.62) ***	(0.25)	(0.64) ***	(0.01) ***	(0.16) ***	(0.25)	(0.14)
Some college	−0.01	−0.61	−1.59	−1.25	0.02	0.86	1.62	0.96
	(0.02)	(0.33) *	(0.16) ***	(0.38) ***	(0.00) ***	(0.09) ***	(0.16) ***	(0.11) ***
College degree	0.2	3.38	−1.53	−6.36	0.05	1.47	2.04	0.76
	(0.02) ***	(0.31) ***	(0.14) ***	(0.36) ***	(0.00) ***	(0.08) ***	(0.14) ***	(0.09) ***
Employed	−0.26	−4.77	0.66	6.78	−0.04	−1.11	−1.15	−0.12
	(0.02) ***	(0.30) ***	(0.13) ***	(0.34) ***	(0.00) ***	(0.08) ***	(0.13) ***	(0.08)
Unable	−0.22	−6.65	−7.13	0.37	0.03	2.36	6.21	5.02
	(0.02) ***	(0.51) ***	(0.43) ***	(0.74)	(0.01) ***	(0.22) ***	(0.41) ***	(0.34) ***
Homeowner	−0.18	−2.86	1.31	5.01	−0.04	−1.21	−1.54	−0.49
	(0.02) ***	(0.34) ***	(0.15) ***	(0.36) ***	(0.00) ***	(0.09) ***	(0.15) ***	(0.09) ***
Married	−0.31	−5.41	1.32	8.18	−0.06	−1.59	−1.78	−0.36
	(0.02) ***	(0.36) ***	(0.15) ***	(0.39) ***	(0.00) ***	(0.09) ***	(0.15) ***	(0.09) ***
Divorced	−0.19	−4.48	−1.22	5.22	−0.02	−0.26	0.25	0.70
	(0.02) ***	(0.40) ***	(0.21) ***	(0.55) ***	(0.00) ***	(0.09) ***	(0.17)	(0.13) ***
Widowed	−0.03	−0.58	0.17	1.00	−0.01	−0.20	−0.26	−0.09
	(0.03)	(0.60)	(0.25)	(0.72)	(0.00) *	(0.13)	(0.24)	(0.16)
Very good health	0.35	8.43	4.71	−5.35	−0.01	−1.23	−3.71	−3.18
	(0.02) ***	(0.23) ***	(0.14) ***	(0.24) ***	(0.00) ***	(0.06) ***	(0.11) ***	(0.10) ***
Poor health	−0.21	−5.65	−3.79	3.43	0.00	0.87	2.81	2.55
	(0.01) ***	(0.27) ***	(0.22) ***	(0.22) ***	(0.00) ***	(0.05) ***	(0.16) ***	(0.16) ***
High blood pressure	−0.61	−13.00	−3.38	14.13	−0.04	−0.50	1.16	2.24
	(0.03) ***	(0.24) ***	(0.14) ***	(0.31) ***	(0.00) ***	(0.06) ***	(0.11) ***	(0.09) ***

All effects on probabilities are multiplied by 100. Asymptotic standard errors are in parentheses. *** *p* < 0.01, ** *p* < 0.05, * *p* < 0.10.

**Table 4:** Marginal effects of explanatory variables on the joint probability of depression and body weight categories for female sample.

	Non-Depressed and				Depressed and			
Variable	Underweight	Normal	Overweight	Obese	Underweight	Normal	Overweight	Obese
Continuous explanatory variables								
Age/10	0.22	3.89	2.49	1.06	−0.06	−2.27	−2.70	−2.64
	(0.01) ***	(0.11) ***	(0.05) ***	(0.10) ***	(0.00) ***	(0.05) ***	(0.05) ***	(0.05) ***
Income	0.04	0.59	0.25	−0.03	−0.00	−0.22	−0.30	−0.33
	(0.01) ***	(0.08) ***	(0.04) ***	(0.08)	(0.00) **	(0.04) ***	(0.04) ***	(0.04) ***
Number of fruits	0.06	0.64	−0.03	−0.50	0.01	0.08	−0.06	−0.20
	(0.01) ***	(0.06) ***	(0.01) ***	(0.05) ***	(0.00) ***	(0.01) ***	(0.01) ***	(0.02) ***
Binary explanatory variables								
Exercise	0.02	0.22	0.01	−0.13	0.00	0.00	−0.04	−0.08
	(0.00) ***	(0.01) ***	(0.00) ***	(0.01) ***	(0.00) ***	(0.00)	(0.00) ***	(0.00) ***
White	−0.40	−5.87	−2.64	−0.03	0.06	2.51	3.16	3.22
	(0.06) ***	(0.54) ***	(0.21) ***	(0.47)	(0.01) ***	(0.20) ***	(0.18) ***	(0.18) ***
Black	−0.84	−10.57	1.91	16.26	−0.17	−4.15	−2.74	0.29
	(0.04) ***	(0.61) ***	(0.28) ***	(0.79) ***	(0.01) ***	(0.17) ***	(0.23) ***	(0.34)
Hispanic	−0.44	−3.81	2.49	7.68	−0.12	−2.90	−2.16	−0.75
	(0.05) ***	(0.68) ***	(0.24) ***	(0.72) ***	(0.01) ***	(0.21) ***	(0.24) ***	(0.29) ***
<High school	0.10	1.24	0.28	−0.42	0.00	−0.22	−0.42	−0.55
	(0.07)	(0.74) *	(0.32)	(0.64)	(0.01)	(0.32)	(0.31)	(0.31) *
Some college	−0.04	−1.69	−2.05	−1.95	0.07	2.00	2.01	1.65
	(0.03)	(0.37) ***	(0.18) ***	(0.33) ***	(0.01) ***	(0.19) ***	(0.17) ***	(0.18) ***
College degree	0.39	2.12	−3.06	−6.18	0.15	3.34	2.34	0.91
	(0.04) ***	(0.37) ***	(0.17) ***	(0.33) ***	(0.01) ***	(0.18) ***	(0.16) ***	(0.17) ***
Employed	−0.34	−2.70	1.45	4.16	−0.09	−1.69	−0.89	0.11
	(0.03) ***	(0.31) ***	(0.14) ***	(0.28) ***	(0.01) ***	(0.14) ***	(0.13) ***	(0.14)
Unable	−0.43	−9.78	−8.08	−5.42	0.19	7.14	8.34	8.04
	(0.04) ***	(0.55) ***	(0.36) ***	(0.49) ***	(0.02) ***	(0.42) ***	(0.36) ***	(0.45) ***
Homeowner	−0.01	0.99	1.67	1.85	−0.06	−1.67	−1.58	−1.20
	(0.03)	(0.36) ***	(0.17) ***	(0.32) ***	(0.01) ***	(0.18) ***	(0.16) ***	(0.17) ***
Married	−0.09	0.48	2.27	3.15	−0.09	−2.32	−2.04	−1.37
	(0.04) **	(0.4)	(0.17) ***	(0.36) ***	(0.01) ***	(0.18) ***	(0.17) ***	(0.17) ***
Divorced	−0.09	−1.61	−1.03	−0.43	0.02	0.92	1.12	1.11
	(0.04) **	(0.47) ***	(0.21) ***	(0.43)	(0.01) **	(0.22) ***	(0.21) ***	(0.22) ***
Widowed	0.25	2.98	0.60	−1.09	0.00	−0.50	−0.98	−1.27
	(0.06) ***	(0.55) ***	(0.23) ***	(0.47) **	(0.01)	(0.23) **	(0.22) ***	(0.21) ***
Very good health	0.98	13.48	4.69	−2.67	−0.02	−3.26	−5.74	−7.47
	(0.04) ***	(0.28) ***	(0.13) ***	(0.21) ***	(0.00) ***	(0.11) ***	(0.13) ***	(0.16) ***
Poor health	−0.60	−9.31	−3.87	1.38	0.00	2.24	4.26	5.89
	(0.03) ***	(0.36) ***	(0.19) ***	(0.15) ***	(0.00)	(0.11) ***	(0.18) ***	(0.28) ***
High blood pressure	−1.26	−14.19	−0.18	11.67	−0.17	−1.86	1.20	4.79
	(0.04) ***	(0.29) ***	(0.14)	(0.30) ***	(0.01) ***	(0.12) ***	(0.13) ***	(0.16) ***

All effects on probabilities are multiplied by 100. Asymptotic standard errors are in parentheses. *** *p* < 0.01, ** *p* < 0.05, * *p* < 0.10.

## Data Availability

The data of this research are publicly available.

## Appendix A

### Appendix A.1. Derivation of Log-Likelihood Function, Marginal Effects, and Average Treatment Effects

The variances of $\left(u_{1},u_{2}\right)$ in Equations (1) and (2) are assumed to be unitary because $y_{1}$ is ordinal and $y_{2}$ is binary. The reduced-form equations are

(A1) $$y_{1}^{*}=x{\prime \Pi }_{11}+z{\prime \Pi }_{12}+w{\prime \Pi }_{13}+v_{1}$$

(A2) $$y_{2}^{*}=x{\prime \Pi }_{21}+z{\prime \Pi }_{22}+w{\prime \Pi }_{23}+v_{2}$$

where $\Pi_{11},\Pi_{12},\Pi_{13},\Pi_{21},\Pi_{22},$ and $\Pi_{23}$ are functions of the structural parameters in Equations (1) and (2), and the composite error vector $v=\left[v_{1},v_{2}\right]\prime$ is distributed as a bivariate normal with zero means, correlation τ, standard deviations $(\omega_{1},\omega_{2}),$ and covariance ${\tau \omega }_{1}\omega_{2}$:

(A3) $$\left[\begin{array}{c} v_{1}\\ v_{2} \end{array}\right]\sim N\left(\left[\begin{array}{c} 0\\ 0 \end{array}\right],\left[\begin{array}{cc} \omega_{1}^{2} & \tau \omega_{1}\omega_{2}\\ \tau \omega_{1}\omega_{2} & \omega_{2}^{2} \end{array}\right]\right)$$

where $\omega_{1}^{2}=\left(1+\gamma_{1}^{2}+2\rho \gamma_{1}\right)/{\left(1-\gamma_{1}\gamma_{2}\right)}^{2},$ $\omega_{2}^{2}=\left(1+\gamma_{2}^{2}+2\rho \gamma_{2}\right)/{\left(1-\gamma_{1}\gamma_{2}\right)}^{2},$ and $\tau =\left[\gamma_{1}+\gamma_{2}+\left(1+\gamma_{1}\gamma_{2}\right)\rho \right]/{\left[\left(1+\gamma_{1}^{2}+2\rho \gamma_{1}\right)\left(1+\gamma_{2}^{2}+2\rho \gamma_{2}\right)\right]}^{1/2}.$

Before constructing the likelihood contribution for the sample observation, first define $h\Pi_{1}=x\prime \Pi_{11}+z\prime \Pi_{12}+w\prime \Pi_{13}$ and $h\Pi_{2}=x\prime \Pi_{21}+z\prime \Pi_{22}+w\prime \Pi_{23},$ where $h={\left[x^{\prime },z^{\prime },w^{\prime }\right]}^{\text{’}}.$ Given

(A4) $$\mathrm{Pr}\left(y_{1}=k,y_{2}=0\right)=\int_{-\infty }^{-h\Pi_{2}}\int_{\xi_{k-1}-h\Pi_{1}}^{\xi_{k}-h\Pi_{1}}f\left(v_{1},v_{2}\right)dv_{1}dv_{2}$$

(A5) $$\mathrm{Pr}\left(y_{1}=k,y_{2}=1\right)=\int_{-h\Pi_{2}}^{\infty }\int_{\xi_{k-1}-h\Pi_{1}}^{\xi_{k}-h\Pi_{1}}f\left(v_{1},v_{2}\right)dv_{1}dv_{2}$$

the joint probability of each body weight category and depression status is

(A6) $$\mathrm{Pr}\left(y_{1}=k,y_{2}=j\right)=\Phi_{2}\left(\frac{\xi_{k}-h\Pi_{1}}{w_{1}},\frac{{\left(-1\right)}^{j+1}h\Pi_{2}}{w_{2}};{\left(-1\right)}^{j}\tau \right)\;-\Phi_{2}\left(\frac{\xi_{k-1}-h\Pi_{1}}{w_{1}},\frac{{\left(-1\right)}^{j+1}h\Pi_{2}}{w_{2}};{\left(-1\right)}^{j}\tau \right),\;j=0,\;1;\;k=0,\;1,\ldots ,K$$

where $\Phi_{2}\left(s,t;r\right)=\mathrm{Pr}\left(S\leq s,T\leq t\right)$ is a bivariate standard normal cumulative function (CDF) with correlation r. The sample likelihood function for an independent sample is the product of (A6) over the sample observations.

To facilitate interpretation of the effects on explanatory variables, marginal effects of explanatory variables on the probabilities of depression and body weight categories are calculated. Specially, for each individual, the probabilities of being depressed or non-depressed are

(A7) $$\mathrm{Pr}\left(y_{2}=j\right)=\Phi_{1}\left(\frac{{\left(-1\right)}^{j+1}h\Pi_{2}}{w_{2}}\right),j=0,1$$

where $\Phi_{1}$ is CDF of the unit normal. Marginal effects of each continuous (binary) explanatory variable can be derived by differentiating (differencing) Equations (A6) and (A7). In addition, to better gauge the effect of depression on each body weight category, we also estimate the average treatment effect of depression, which is the average of

(A8) $$TE_{k}=\mathrm{Pr}\left(y_{1}=k|y_{2}=1\right)-\mathrm{Pr}\left(y_{1}=k|y_{2}=0\;\right),k=0,1,\ldots ,K$$

over the sample. For statistical inference, standard errors of the marginal and treatment effects can be derived by the delta method.

### Appendix A.2. Additional Tables

**Table A1:** Variable definitions and sample statistics.

Variable	Definition	Male	Female
Endogenous variables			
Body Weight	Ordinal indicator of body mass index (1–4)	3.04	2.89
		(0.76)	(0.85)
Depression	Have depressive symptoms	0.11	0.23
Continuous explanatory variables			
Age	Age in years	55.60	56.57
		(16.85)	(16.36)
Income	Annual household income level (1–8)	6.63	6.15
		(1.81)	(2.03)
Number of fruits	Total fruits consumed per day	1.53	1.63
		(2.16)	(2.00)
Exercise	Number of times doing physical activity during last 30	15.99	16.11
	days	(12.05)	(11.75)
Binary explanatory variables (yes = 1, no = 0)			
White	Race is White	0.80	0.79
Black	Race is Black	0.05	0.07
Hispanic	Race is Hispanic	0.07	0.07
Other race	Other race (reference)	0.07	0.06
Base	Do not have high school diploma	0.04	0.04
High school	Has a high school diploma or GED (reference)	0.21	0.20
Some college	Has some college but not a Bachelor’s degree	0.26	0.29
College degree	Has a Bachelor’s degree or above	0.48	0.47
Employed	Employed	0.60	0.49
Unable	Unable to work	0.03	0.05
Home owner	Home owner	0.78	0.77
Married	Married	0.63	0.54
Divorced	Divorced	0.11	0.14
Widowed	Widowed	0.05	0.14
Single	Single, separate, or unmarried couple (reference)	0.20	0.17
High blood pressure	Have been told by a doctor that they have high blood pressure	0.42	0.35
Very good health	Self-report very excellent or very good health status	0.58	0.59
Good health	Self- report good health status (reference)	0.30	0.28
Poor health	Self-report fair or poor health status	0.12	0.12
Sample size		71,603	71,034

Note: Standard deviations are in parentheses. Income is the annual household income reported as categories from 1 to 8: 1 = less than $10,000, 2= $10,000 to $15,000, 3 = $15,000 to $20,000, 4 = $20,000 to $25,000, 5 = $25,000 to $35,000, 6 = $35,000 to $50,000, 7 = $50,000 to $75,000, and 8 = $75,000 or more.

**Table A2:** Full information on the maximum-likelihood estimation of the simultaneous equation system.

	Male		Female	
Variable	Depression	Body Weight	Depression	Body Weight
Depression (γ_1_)		0.472 (0.015) ***		0.485 (0.012) ***
Body weight (γ_2_)	0.407 (0.029) ***		0.317 (0.028) ***	
Constant	−0.396 (0.072) ***	1.690 (0.038) ***	0.297 (0.079) ***	1.823 (0.041) ***
Age/10	−0.194 (0.005) ***	0.077 (0.005) ***	−0.260 (0.005) ***	0.090 (0.005) ***
Exercise	0.000 (0.001)	−0.002 (0.000) ***	−0.002 (0.001) ***	−0.004 (0.000) ***
Income	−0.063 (0.004) ***	0.051 (0.003) ***	−0.027 (0.004) ***	0.005 (0.003)
White	0.201 (0.025) ***	−0.025 (0.019)	0.324 (0.024) ***	−0.082 (0.019) ***
Black	−0.201 (0.037) ***	0.237 (0.027) ***	−0.402 (0.033) ***	0.547 (0.025) ***
Hispanic	−0.168 (0.033) ***	0.211 (0.025) ***	−0.290 (0.030) ***	0.295 (0.024) ***
<High school	0.076 (0.032) **	−0.118 (0.024) ***	−0.035 (0.033)	−0.006 (0.025)
Some college	0.200 (0.019) ***	−0.100 (0.014) ***	0.201 (0.017) ***	−0.105 (0.013) ***
College degree	0.320 (0.018) ***	−0.269 (0.013) ***	0.285 (0.017) ***	−0.262 (0.013) ***
Employed	−0.215 (0.017) ***	0.243 (0.012) ***	−0.129 (0.014) ***	0.162 (0.011) ***
Unable	0.522 (0.032) ***	−0.137 (0.027) ***	0.665 (0.027) ***	−0.263 (0.024) ***
Home owner	−0.235 (0.017) ***	0.208 (0.013) ***	−0.161 (0.015) ***	0.093 (0.013) ***
Married	−0.305 (0.019) ***	0.311 (0.014) ***	−0.222 (0.017) ***	0.149 (0.014) ***
Divorced	−0.021 (0.023)	0.130 (0.018) ***	0.104 (0.020) ***	−0.035 (0.017) **
Widowed	−0.044 (0.034)	0.040 (0.025)	−0.081 (0.024) ***	−0.017 (0.019)
Very good health	−0.376 (0.018) ***		−0.479 (0.017) ***	
Poor health	0.258 (0.016) ***		0.334 (0.016) ***	
Number of fruits		−0.012 (0.001) ***		−0.016 (0.002) ***
High blood pressure		0.339 (0.013) ***		0.371 (0.012) ***
μ_2_, ξ_2_		1.666 (0.020) ***		1.754 (0.015) ***
μ_3_, ξ_3_		2.784 (0.027) ***		2.592 (0.019) ***
ρ		−0.745 (0.022) ***		−0.648 (0.024) ***
Log likelihood	−96,798.463		−109,884.58	

Asymptotic standard errors are in parentheses. *** *p* < 0.01, ** *p* < 0.05.
